# Cholestasis induced by bile duct ligation promotes changes in the intestinal microbiome in mice

**DOI:** 10.1038/s41598-019-48784-z

**Published:** 2019-08-23

**Authors:** Raul Cabrera-Rubio, Angela M. Patterson, Paul D. Cotter, Naiara Beraza

**Affiliations:** 1 Teagasc Food Research Centre, Moorepark, Fermoy, Co, Cork, Ireland; 2 APC Microbiome Institute, University College Cork, Co, Cork, Ireland; 3 Gut Microbes and Health Institute Strategic Programme, Quadram Institute, Norwich Research Park, Norwich, UK

**Keywords:** Microbiology, Cholestasis

## Abstract

Increasing evidence point to the relevance of intestinal disfunction and changes in the microbiome composition during chronic liver disease. More specifically, recent studies have highlighted that cholestatic diseases associate with a reduction in the microbiome diversity in patients. Still, the dynamics of the changes in the microbiome composition observed, as well as their implication in contributing to the pathogenesis of this disease remain largely undefined. Hence, experimental mouse models resembling the human pathogenesis are crucial to move forward our understanding on the mechanisms underpinning cholestatic disease and to enable the development of effective therapeutics. Our results show that the bile duct ligation (BDL) experimental model of cholestasis leads to rapid and significant changes in the microbiome diversity, with more than 100 OTUs being significantly different in faecal samples obtained from WT mice at 3 days and 7 days after BDL when compared to control animals. Changes in the microbial composition in mice after BDL included the enrichment of *Akkermansia*, *Prevotella*, *Bacteroides* and *unclassified Ruminococcaceae* in parallel with a drastic reduction of the presence of *Faecalibacterium prausnitzii*. In conclusion, our results support that bile duct ligation induces changes in the microbiome that partly resemble the gut microbial changes observed during human cholestatic disease.

## Introduction

Our intestine is home to trillions of microorganisms, including bacteria, archaea, fungi and virus, which can be regarded as a superorgan referred to as the gut microbiota, while the gut microbiome refers to the microbial genome. There is increasing evidence of the close relationship between the microbiome and human health. Indeed, a number of chronic diseases ranging from atherosclerosis^1^, diabetes^2^, metabolic syndrome and obesity^3^ to neurodegenerative disorders^4^ have recently been associated with disturbances in the intestinal bacterial composition.

Chronic liver disorders have also been associated with changes in gut microbiome composition and the disruption of the intestinal barrier^3,5–8^. The ‘leaky gut’ hypothesis proposes that intestinal bacteria, endotoxin or other bacterial products translocate from the permeable gut into the liver via the porta circulation, triggering hepatic inflammation and fibrosis further contributing to the progression of chronic liver disease^5,7^.

Recent studies have further supported the association between changes in the microbiome and the progression of cholestatic liver disease in patients^9–14^. Cholestasis occurs when the natural flux of bile acids from the liver to the gut is impaired, mainly as a result of damage in the biliary ducts, causing accumulation of toxic bile acids in the liver that promote cell death, inflammation, fibrogenesis and ultimately cirrhosis and cancer^15^. Cholestasis results from different primary aetiologies but also is a frequent secondary event occurring during most chronic liver diseases^16^. In adults, primary biliary cholangitis (PBC) and primary sclerosing cholangitis (PSC) are the main aetiologies causing cholestasis. The available treatments for PBC patients are limited to ursodeoxycholic acid (UDCA) and the recently licenced obeticholic acid (OCA) as a second-line approach^17,18^. Still, a proportion of these patients remain unresponsive to treatment. In this line, there is currently no available treatment for PSC patients and liver transplantation is the only curative option, which has obvious shortcomings including scarcity of donors and a high incidence of recurrence^19^. Overall, these highlight the urge to find new approaches to treat cholestatic patients.

The use of experimental animal models that resemble human disease is crucial to define the mechanisms underpinning the pathogenesis of cholestasis, including the potential role of the microbiome in the progression of the disease.

Based on the complexity of the multiple molecular events undergoing during cholestasis, involving interactions between different liver cell types and the gut-microbiome-liver axis, the current consensus is that there is not a unique animal model fully recapitulating human cholestatic disease^20^. While concurring with this conclusion, we here demonstrate that bile duct ligation (BDL) is a plausible model to investigate the functional relevance of the microbiome in the pathogenesis of cholestasis. Thus, our in-depth 16S rRNA-based analysis shows that BDL induces significant changes in the intestinal microbiome composition of mice, characterised by a reduction in diversity at early stages and changes in the abundance of particular bacteria, partially resembling the observations described during human cholestasis.

## Results

### Cholestasis induced by bile duct ligation promotes liver injury and inflammation that associates with increased intestinal permeability

**Figure 1:**
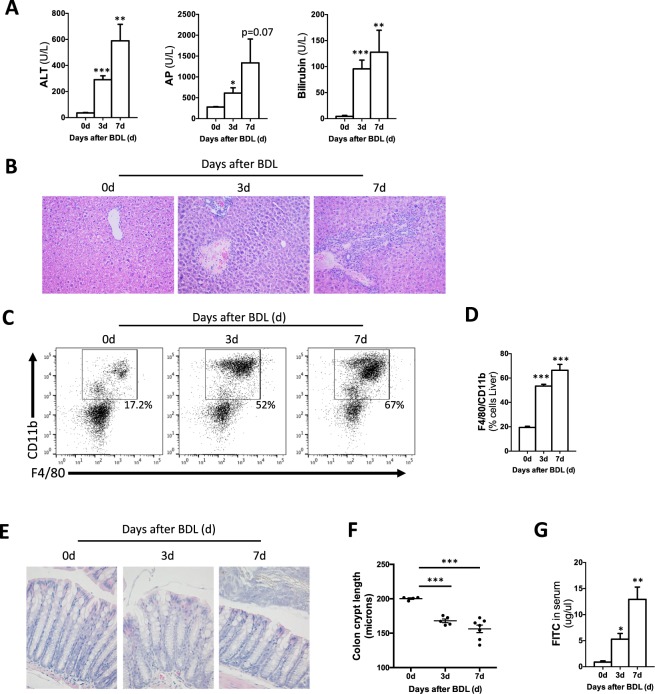
Effect of BDL in the liver and the intestine. (**A**) Serum liver damage markers ALT, AP and Bilirubin showed increased levels in WT mice 3days and 7 days after BDL. (**B**) Haematoxylin and Eosin staining on liver sections showing profuse necrosis in livers from mice after BDL. (**C**,**D**) FACS analysis of immune cells isolated from livers showing increased numbers of CD11b/F4/80 cells in livers after BDL. (**E**) Histopathological analysis of colon sections showing tissue damage after BDL and (**F**) shortening of colon crypts (in microns). (**G**) Detection of FITC in serum samples from mice gavaged with FITC-Dextran 3 hours before sacrifice showing increased intestinal permeability. *P < 0.05, **P < 0.01, ***P < 0.001 [Control *vs* BDL].

To induce cholestasis, we performed bile duct ligation (BDL) in mice that were sacrificed at 3 days and 7 days after surgery. The liver damage serum marker alanine aminotransferase (ALT), and the surrogate markers of cholestasis alkaline phosphatase (AP) and bilirubin were significantly increased at 3 and 7d after BDL when compared to control mice (Fig. 1A). Histopathological analysis on liver sections by H&E staining confirmed the induction of liver damage after BDL as evidenced by the presence of wide areas of necrosis at 3d and 7d after surgery (Fig. 1B). Macrophages, as part of the innate immune response, are the first line of response against pathogens, including bacterial products (pathogen-associated molecular patterns; PAMPs) that translocate from the intestine during cholestasis. FACS analysis on isolated immune cells showed a significant increase in the number of liver infiltrated CD11b^+^/F4/80^+^ macrophages (Fig. 1C,D).

BDL was associated with changes of the intestinal architecture as observed histologically, including histopathological disruption in the colon with the shortening of crypt length (Fig. 1E,F), epithelial cell atrophy, and increase in intraepithelial lymphocytes and immune cell infiltrates (Fig. 1E). Changes in the epithelial cell population in colons from BDL 7d mice were more pronounced, with cellular degradation of luminal epithelial cells characterised by vacuoles, blebbing and apoptotic bodies (Fig. 1E).

These structural changes were also associated with increased intestinal permeability in WT mice at 3 and 7 days after BDL, as evidenced by the presence of circulating FITC in serum samples after gavage administration prior to sacrifice (Fig. 1G).

### Bile duct ligation promotes early changes in the intestinal microbiome

Microbiome analysis was facilitated by 16S rRNA analysis. After quality filtration and length trimming, an average of 116559 (±37266 SD) high-quality 16S rRNA sequences were generated per sample and grouped by sample type, i.e., Control: Control mice, BDL 3d: Bile duct ligation (after 3 days), BDL 7d: Bile duct ligation (after 7 days).

**Figure 2:**
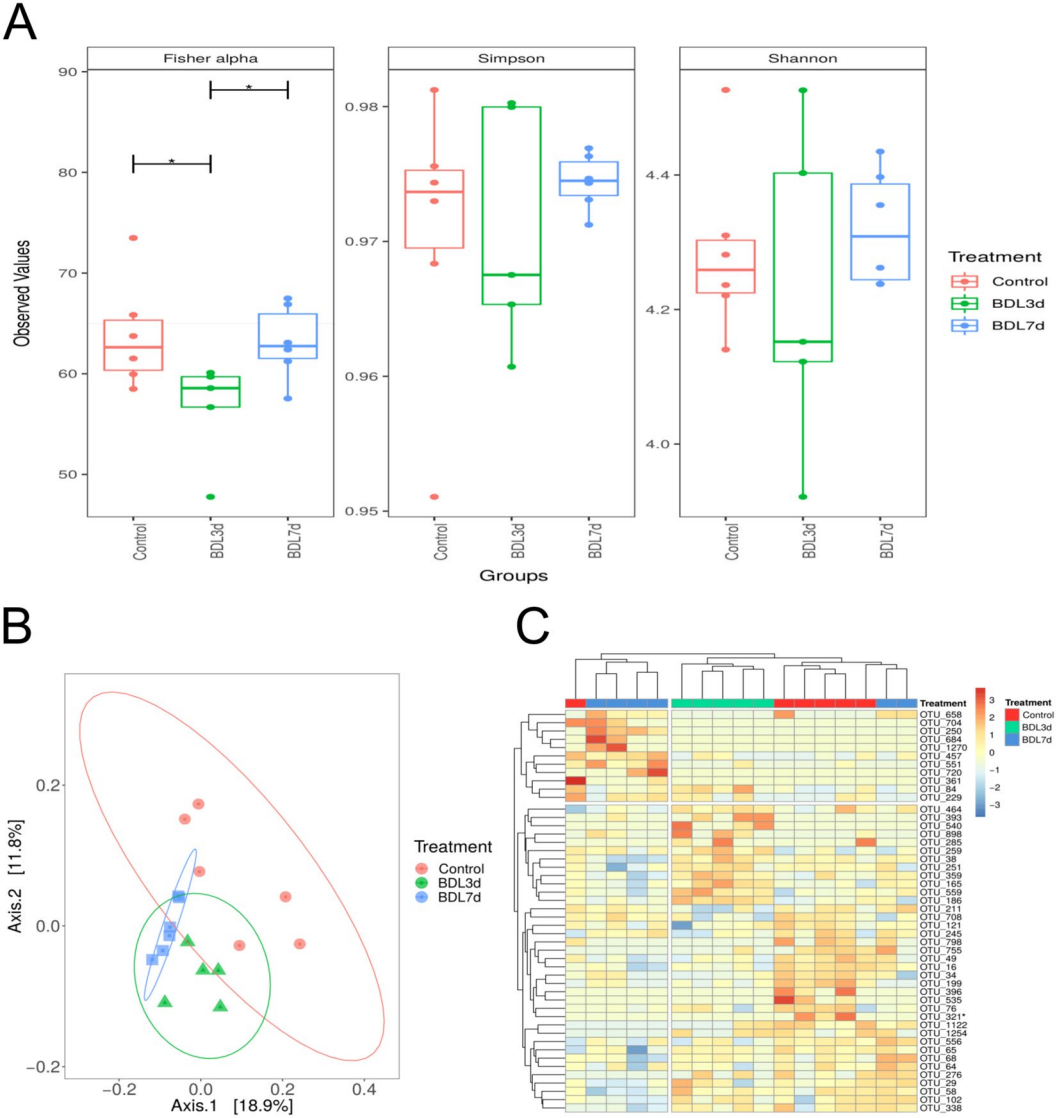
Effects of BDL treatment on the mice microbiome. (**A**) Fisher, Shannon and Simpson index alpha diversity measures of bacterial number and distribution by group. (**B**) Principal Coordinate analysis (PCoA) of unweighted Unifrac distances of generated 16s rRNA sequences highlighting the change in microbial composition along the BDL treatment. (**C**) Bray-Curtis heatmap of beta diversity correlations between samples with OTUs significance (p < 0.05). Axes indicate the individual sample per row (right x-axis) and your relation with OTUs in the column (bottom y-axis). *P < 0.05 [Control *vs* BDL; BDL 3d vs BDL 7d].

Fisher’s alpha diversity index showed significant differences between Control vs BDL 3d and BDL 3d vs BDL 7d groups (P < 0,05; Fig. 2A). Shannon and Simpson alpha diversity index did not change significantly over time (Fig. 2A). Notably, significant differences (P = 0.001) in beta diversity, as shown by Principal Coordinates Analysis (PCoA) were apparent after BDL in both 3d and 7d groups (Fig. 2B). Here, UniFrac unweighted distances were calculated by pairwise comparison of the groups of samples, and significant differences were detected when the following comparisons were done; Control vs BDL 3d (P < 0.013), Control vs BDL 7d (P < 0.006) and BDL 3d vs BDL 7d (P < 0.006) (Fig. 2B).

We found a number of OTUs that were significantly different (P < 0.05) when all groups were compared, showing separation as plotted on a heatmap (Fig. 2C). Further analysis showed significant differences in some OTUs of interest assigned to genus with lower proportions of *Faecalibaculum (including F prausnitzii as identified by OTU 321)* in the BDL 3d samples, and a transient decrease in the genus Akkermansia in the BDL 3d sample group that increases very abruptly in the BDL 7d sample where it shows high values (Fig. 2C). Higher proportions of *Clostridium* (including OTUs 38, 165, 251, 359, 393, 559) in both BDL groups (BDL 3d and BDL 7d) were also observed when compared to control samples.

**Figure 3:**
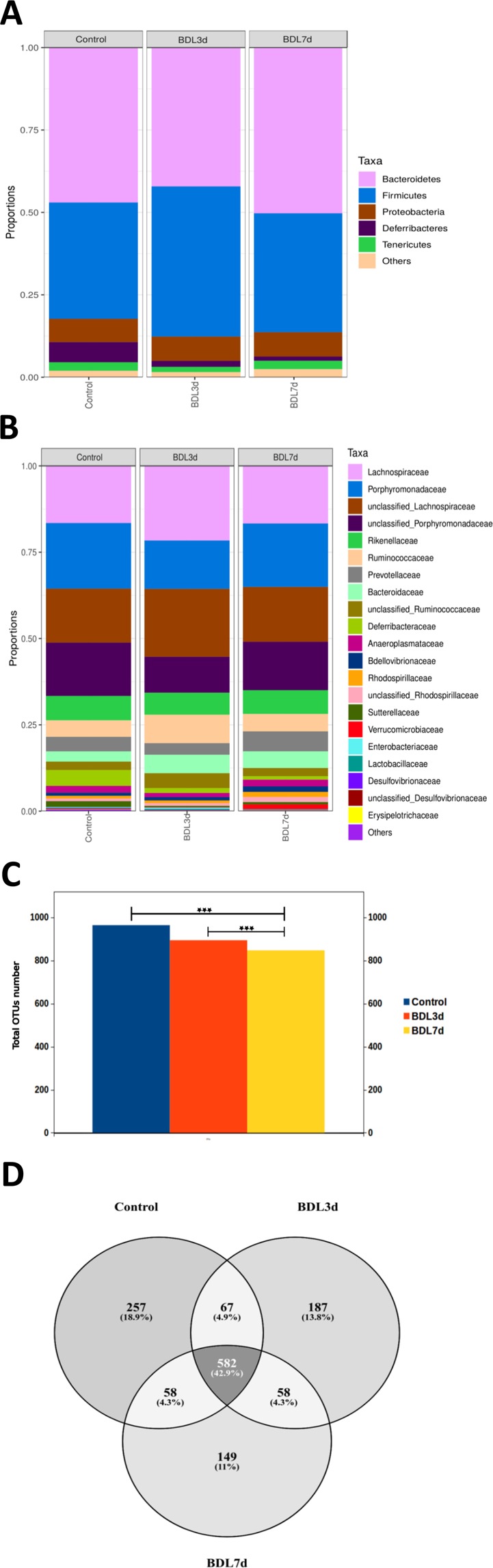
Effects of BDL treatment on bacterial composition. (**A**) Phylum level bacterial composition by group. (**B**) Family level bacterial composition by group. (**C**) OTUs number with possible number of bacteria and distribution by group. (**D**) Venn diagram showing percentage of exclusive OTUs per groups. *P < 0.05, ***P < 0.001 [Control *vs* BDL; BDL 3d vs BDL 7d].

With respect to taxonomy, an average of 613.12 (±66.72 SD) OTUs were obtained, being assigned mainly to the phyla *Firmicutes*, *Bacteroidetes* and *Proteobacteria* (Fig. 3A, Supplemental Fig. S1). The differences were already at this level, observing higher levels in the ratio *Firmicutes*/*Bacteroidetes* (ratio F/B) in the BDL 3d samples (ratio F/B = 1.082) compared to the group of control samples (ratio F/B = 0.75) and finally the BDL 7d samples (ratio F/B = 0.72) recover the initial ratio.

Further analysis at the family level supported the impact of BDL in promoting changes in the microbiome composition as shown in Fig. 3B, that evidences change in the *Porphyromonadaceae*, *Lachnospiraceae*, *Rikenellaceae*, *Ruminococcaceae* and *Prevotellaceae* families at 3d and 7d after BDL (Fig. 3B, Supplemental Fig. 1B).

The impact of bile duct ligation in promoting changes in the microbiota was also evidenced by a statistically significant decrease in proportions of more than 100 OTUs (117 OTUs) when mice subjected to BDL were compared individually to control animals (Fig. 3C). More specifically, as shown in the Venn diagram, 257 OTUs (18.9%) were found in the control group, 187 OTUs (13.8%) in BDL 3d group and 149 OTUs (11%) in BDL 7d group (Fig. 3D). The core microbiome was composed of 582 OTUs (42.9%). Paired comparisons highlighted 67 common OTUs (4.9%) to control and BDL 3d samples only, 58 OTUs (4.3%) common to control and BDL 7d groups only and another 58 OTUs (4.3%) common to the BDL 3d and BDL 7d groups only (Fig. 3D).

**Figure 4:**
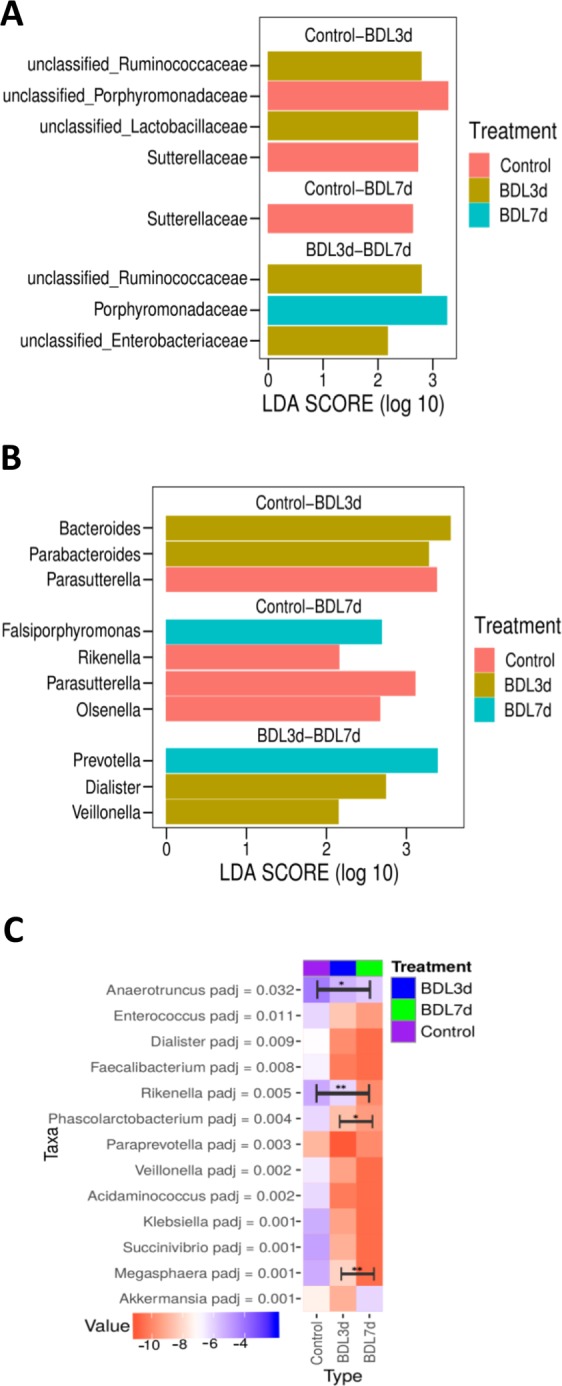
Statistically significant taxonomic changes in BDL treatment. (**A**) Linear Discriminant Analysis (LDA) Effect Size (LefSe) on 16srRNA sequencing data serts showing differences at family level and (**B**) genus level. **(C)** Negative binomial GLM fitting and Wald statistics analysis of differences at genus level. All bacteria show in LDA graphs are statistically significant. *P < 0.05, **P < 0.01 [Control *vs* BDL 3d; Control *vs* BDL 7d; BDL 3d vs BDL 7d].

Assessment with linear discrimination analysis (LDA) effect size >2, LefSe (Wilcoxon test) at family level showed statistically significant differences when respective pairs of groups were directly compared. More specifically, a significant reduction in *unclassified Porphyromonadaceae* (p < 0.05) and *Sutterellaceae* (p < 0.05), as well as an increase of *unclassified Ruminococaceae* and *unclassified Lactobacillaceae* in the BDL 3d group compared to control samples (Fig. 4A). In addition, we found a reduction of *Sutterellaceae* (p < 0.05) in BDL 7d samples when compared to the control group. Finally, when the BDL 3d and BDL 7d groups were compared, we found greater proportions of *unclassified Ruminococcaceae* (p < 0.05) and *unclassified Enterobacteriaceae* in BDL 3d group and lower presence of *Porphyromonadaceae* (p < 0.05), which was enriched in BDL 7d samples with respect to the BDL 3d samples (Fig. 4A).

At genus level in BDL 3d samples, LDA showed statistically significant increase in the genus *Bacteroides* (p < 0.05) and *Parabacteroides* (p < 0.05), whereas proportions in the genus *Parasutterella* (p < 0.05) were reduced when compared to controls. Also, we found statistically significant increase in the genus *falsiporphyromonas* (p < 0.05), whereas proportions in the genus *Rickenella* (p ≤ 0.05), *Parasutterella* (p < 0.05) and *Olsenella* (p < 0.05) were reduced in BDL 7d samples when compared to controls. Abundance of the *Prevotella* (p < 0.05) genus was increased in samples from BDL 7d mice when compared to BDL 3d samples, while *Dialister* (p < 0.05) and *Veillonella* (p < 0.05) were more abundant in BDL 3d compared to BDL 7d (Fig. 4B).

In addition, we performed a differential expression analysis based on the Negative Binomial distribution to observe statistical changes at the genus level (p < 0.05) with a different approach. We found an increase in the genus *Akkermansia* at 7days after BDL with respect to the other groups of samples (Fig. 4C). The remainder genera shown in Fig. 4C (*Anaerotruncus*, *Enterococcus*, *Dialister*, *Faecalibacterium*, *Rikenella*, *Phascolarctobacterium*, *Paraprevotella*, *Veillonella*, *Acidaminococcus*, *Klebsiella*, *Succinivibrio* and *Megasphaera*) had a statistically significantly lower proportion in the BDL 3d and BDL 7d groups compared to control samples.

## Discussion

In this study we demonstrate that cholestasis induced by the ligation of the bile duct (bile duct ligation; BDL) in mice promotes significant changes in microbiome composition, mainly characterised by a reduction in beta diversity.

The role of the microbiome during the pathogenesis of cholestatic liver disease is still poorly understood. Recent studies described that cholestatic patients with PSC have an overall less diverse microbiome composition. The changes described at genera level differ among studies^9–12^ and include the enrichment in *Veillonella*, *Akkermansia*, *Ruminococcaceae (undefined genus)* and *Clostridium*^9^ and the decrease in *Prevotella*^10^, overall supporting the association between changes in the microbiome and cholestatic disease. Few studies have investigated the composition of the microbiome in PBC patients and importantly show some similarities with those observed in PSC^13,14^.

Previous studies showing the benefits of antibiotics in improving liver function in cholestatic patients point to an active role of the microbiome in mediating liver injury during cholestasis^21^. Although these antibiotic-based treatments have transient benefits in cholestasis patients, antibiotics can induce chronic cholestasis and DILI (reviewed in^22^). In addition, the long-lasting and severe effects that antibiotics have in the microbiome composition must be carefully considered when used in the clinical practice^23,24^. Ultimately, the numerous side effects, the recurrence when treatment is abandoned and the serious issues of antibiotic-induced microbiota disturbances observed in other pathologies, underline the pressing need to find alternative strategies to modulate the microbiome as a therapeutic approach. Thus, to better understand how changes in the microbiome composition and functionality impact on disease progression will enable improved therapeutics for cholestatic patients.

For these reasons, valid experimental animal models resembling the pathogenesis of human cholestasis are crucial to gain insight into the mechanisms underlying the progression of this disease and to test therapeutic approaches to treat this condition. Currently, there are different cholestasis experimental animal models available^20^, though the consensus is that there is not an ideal model resembling all features of human cholestatic disease. Moreover, there are very limited available studies focused on characterising the microbiome composition during experimental cholestasis and these studies have shown conflicting results^25,26^. While Fouts *et al*.^26^ described that BDL did not alter the microbiome in mice, Alaish *et al*.^25^ described changes in Bl6/C57 mice at late stages after BDL (14 days) that the authors discussed to be associated with advanced fibrogenesis (cirrhosis).

Our current study supports that indeed, BDL promotes changes in the composition of the microbiome and that changes already occur at early stages of the disease, supporting the active role of the microbiome in the pathogenesis of this condition. The discrepancy of our results with the Fouts *et al*. study, where no changes in microbial composition were found after BDL, may be better explained by the sample origin; cecal and mucosal *vs* faecal samples used in our present work. Also, the sequencing methodology used in that study was different that the one we have applied. Fouts and colleagues used pyrosequencing^26^ while we used Illumina sequencing in our work, which provides a higher number of sequences per sample and greater depth of sequences corresponding to the hypervariable zone V3-V4, as opposed to the hypervariable V1–V3 region targeted in the Fouts *et al*. work^26^. In addition, microbiome analysis was performed at later timepoints (10 days) after BDL, while our analyses comprised earlier timepoints after surgery; 3 and 7 days after BDL. Intriguingly, the Alaish *et al*.^25^ study described differences at later timepoints after BDL (14d), though it is beyond the scope of our study to explain the discrepancy with the Fouts study^26^.

Interestingly, our results are in line with those found in other models of cholestatic disease like the genetically modified Mdr2−/− mice^27^, which show certain similarities in bacterial composition to mice undergoing BDL, including enrichment of Lactobacillus, Prevotella and Clostridiaceae as we show in the present study.

More importantly, some of the changes found during human disease are replicated in mice after BDL as we observed a significant enrichment of *Akkermansia*, in line with the observations in the study by Kummen and colleagues^9^. The abundance of Akkermansia, a mucus degrading bacteria, has been described to negatively correlate with obesity in patients^28^ and mice^29^ although it is enriched in stool samples from alcohol-induced steatohepatitis in mice^30^. The mucus degrading activity of Akkermansia leads to enhanced mucus production^31^, which could determine its beneficial or detrimental impact on the different disease settings by regulating the thickness of the mucus layer. Interestingly, the role of mucins (the glycoproteins that compose the mucus) during liver disease remains controversial as mucin deficiency protects from alcohol induced hepatitis in mice^32^, while enhanced mucus thickness relates to improved liver function in other study of experimental ALD^33^. Interestingly, increased mucus thickness has been observed after BDL^34^ pointing to a role of mucins and likely mucus-degrading bacteria during cholestasis.

In this line, we found increased abundance in an unidentified genus of *Ruminococcacceae* in mice at 3days after BDL. Ruminocccaceae are mucin-degrading bacteria that can both influence mucus thickness and glycan composition^35^. Interestingly, it has been recently described that a specific strain of *Ruminococcacea* (*R*. *gnavus*) modifies sialic acid in the mucus thus limiting the availability of feed to other bacteria species^35^. This mechanism is particularly relevant in IBD, where an increase in *R gnavus* has been described^35^. Future research is guaranteed to determine whether the *Ruminococcacea* species enriched during cholestasis have the relevant enzymatic machinery to modify the mucus composition and how this may impact on the mucus layer and the overall intestinal permeability and barrier function. Interestingly, *Ruminococcaceae* have been found to be enriched in samples from fibrotic patients with NAFLD/NASH^36,37^, supporting their role in the pathogenesis of other chronic liver diseases that also associate with impaired intestinal barrier function.

As well, in those studies it was shown that NALFD/NASH in patients associated with increased abundance of *Bacteroides* abundance^36,37^. Interestingly, *Bacteroides* (enriched in BDL 3d) increase their abundance when the intestinal pH changes from acid to more basic, in detriment of bacteria (e.g. *Faecalibacterium prausnitzii*) that produce butyrate, a short chain fatty acid with well-known beneficial functions in the intestine^38,39^. The regulation of bacterial communities by pH^40^ could be relevant in the context of cholestasis where the presence of bile acids in the intestine decreases.

Accordingly, we found a significant reduction in the abundance of *F*. *prausnitzii* in mice after BDL. As a butyrate-producer, *F*. *prausnitzzi* has anti-inflammatory properties^41^ and its lower abundance after BDL may contribute to the increased permeability and loss of intestinal barrier function during cholestasis. The depletion of *F prausnitzii* in IBD patients^3,41,42^, including ulcerative colitis^43^, a condition that closely relates to PSC, further supports the impact of the loss of this beneficial bacteria in intestinal function and its potential as a therapeutic option to treat these patients^44^.

*Clostridium* was increased in BDL 7d samples, which is in agreement with what was found in PSC patients^9^ as well as in PBC patients^13^. In addition to cholestasis, increased abundance of Clostridium has been linked to IBD and more specifically, C difficile infection is a common complication of IBD^45^. Interestingly, Clostridium, as well as *Bacteroides*, associate with increased LPS and liver injury in a mouse model after high fat diet^46^. In line with this, Enterobacteriaceae are also gram negative-LPS producing bacteria with pro-inflammatory activity, which abundance is increased in NASH patients when compared to obese^47^ and in NAFLD/cirrhotics^48^. Thus, the increased abundance of Enterobacteriacea, Clostridium and Bacteroides found after BDL may contribute to the overall progression of liver disease by promoting LPS-induced liver injury and inflammation.

Following the observations of Quraishi *et al*.^10^, in PSC patients we found reduction of *Prevotella* in mice at 3days after BDL though the abundance was increased at the later timepoint (7d). Increased presence of *Prevotella* has been found in cirrhotics^49^ but most interestingly, *Prevotella* is distinctively found in the oral cavity. Changes in the composition of the oral microbiome and the colonisation of the lower intestinal tract by oral-specific bacteria has been related to cirrhosis^49^. While livers obtained from mice at 7days after BDL do not show evidences of cirrhosis (although fibrosis is obvious in these animals^50^), the increase in *Prevotella* at this timepoint may be an early indicator of the colonisation of the intestinal microbiome with oral-related bacteria. Nevertheless, the reduced presence of other oral-related bacteria such as *Veillonella*, *Megasphera* and *Dialister* in BDL mice may indicate that this colonisation is gradual in time and likely occurring at later stages of the disease, as BDL 7d showed enrichment of *Veillonela* and *Dialister* with respect to BDL 3d samples.

Interestingly, our work provides improved statistical results that demonstrate the reduction of the microbiome diversity at early stages of cholestasis, while these do not reach statistical significance at later timepoints. This is in accordance with previous studies where the diversity of the microbiome was significantly lower in NAFLD patients without fibrosis, whereas the diversity in patients with advanced fibrosis was ‘restored’ to comparable levels to healthy individuals^48^. In line with this, Chen *et al*.^51^ found a similar Chao index in cirrhotic patients and a non-significant Shannon index when compared to healthy controls. Importantly, the composition of the microbiome was altered in all these patients regardless of the lack of statistically significant changes in diversity, supporting that significant changes in microbial composition does not necessarily reflect in the overall diversity index. A possible explanation to these events could be the so-called ‘Anna Karenina’ principle where stress promotes a stochastic but not deterministic changes in the microbiome composition^52^. In addition, the emergence of opportunistic pathogens at later stages of liver disease, which remain in low abundance in the context of health when commensal bacteria dominate the intestinal microbiome may also result in an increased diversity.

Overall, our results support the suitability of the BDL experimental model as an effective tool to investigate the role of the microbiome in the progression of cholestasis as well as a platform to test therapeutic approaches focused on the modulation of the microbiome, applicable for human disease. Future research will be essential to define the causality of the changes in the microbiome as contributors of the progression of cholestatic disease.

## Materials and Methods

### Experimental procedures in animals

*C57/B6J* mice were generated at the Disease Model Unit (University of East Anglia, UK). All experimental procedures were conducted in male mice from 8–12 weeks of age and were approved by the Animal Welfare and Ethical Review Body (AWERB, University of East Anglia, Norwich, UK). All experiments were performed following the guidelines of the National Academy of Sciences (National Institutes of Health publication 86-23, revised 1985) and were conducted within the provisions of the Animals (Scientific Procedures) Act 1986 (ASPA) and the LASA Guiding Principles for Preparing for and Undertaking Aseptic Surgery (2010) under UK Home Office approval. Cholestasis was induced by ligating the common bile duct (bile duct ligation; BDL) as we previously described^50,53^.

### Histopathological analysis of liver and intestinal tissue

Liver and intestinal tissues were embedded in paraffin and further sectioned, dewaxed, hydrated and stained with H&E for pathological analysis.

### Flow cytometry

Immune cells were isolated from liver tissues after digestion with collagenase and successive washes and Percoll gradient. Isolated immune cells were stained with CD45-APC-Cy7 (BD), CD11b-PE (BD) and F4/80-FITC (Myltenyi) antibodies. Flow cytometry analysis was performed using BD LSRFortessa and analysed using FlowJo software.

### DNA Isolation, sequencing and bioinformatic analysis (16S rRNA gene) from faecal samples

Fresh faecal pellets were obtained from mice immediately before culling and snap frozen in liquid nitrogen. The DNA extraction and sequencing was done according to Gough *et al*.^54^. The fastq sequences (from Illumina MiSeq platform (2 × 250 bp)) were filtered on the basis of quality (removal of low quality nucleotides at the 3′ end) and length (removal of sequences with less than 200 nt) with prinseq^55^, Paired-end reads with a minimum overlap of 20 bp were joined using Fastq-join^56^.

The filtered sequences were matched at operational taxonomic unit (OTU; with 97% identity level) using UPARSE-OTU algorithm with usearch v7.0 program^57^ and remove chimeric OTUs against gold database. The taxonomic assignment of these OTUs was obtained against the Ribosomal database project (RDP)^58^.

### Statistical analysis

Downstream analyses and graphical outputs the 16S rRNA gene were generated with different packages in R^59^. Alpha diversity was computed with vegan R package^60^, and was visualized via the phyloseq package^61^, also we use ANOVA and t-test to calculate the significance in the analysis of alpha-diversity index. For beta diversity analysis, dissimilarity matrix between samples was calculated with Unifrac Unweight method^62^, studying the effects of treatment and time on microbiota composition variability between samples through permutational multivariate analysis of variance (PERMANOVA) with the adonis function and with pairwise adonis with FDR corrections. For heatmap we conducted a multivariate analysis using the mvabund package (3.9.3)^63^, using the function anova.manyglm, adjusted for multiple testing, multivariate and univariate results for each OTU were obtained. Linear Discriminant Analysis (LDA) Effect Size (LefSe)^64^ algorithm with LDA effect size threshold of 2 (on a log^10^ scale) was applied after agglomerating data to family and genus level for evidencing potential biomarkers linked. Statistical differences between multiple samples at Phylum, Family and Genera-level were estimated by Kruskal-Wallis or Mann–Whitney *U*-test or Negative Binomial distribution^65^ by adjusted for multiple testing according to the method of Benjamini and Hochberg^66^. Significance (p < 0.05) was based on the corrected p-values.

## Supplementary information

Supplemental information
