# Supplementary material for: Cholestasis induced by bile duct ligation promotes changes in the intestinal microbiome in mice
Source: Sci Rep. 2019 Aug 23;9:12324. doi: 10.1038/s41598-019-48784-z (PMC6707139; doi:10.1038/s41598-019-48784-z)
Supplement: Supplementary file 1 — Supplemental information [file 41598_2019_48784_MOESM1_ESM.pdf]

Cholestasis induced by bile duct ligation promotes changes in the intestinal microbiome in mice

Raul Cabrera-Rubio[1, 2], Angela M Patterson[3], Paul D Cotter[1,2], Naiara Beraza[3]

Supplemental material for Cabrera-Rubio et al.

A

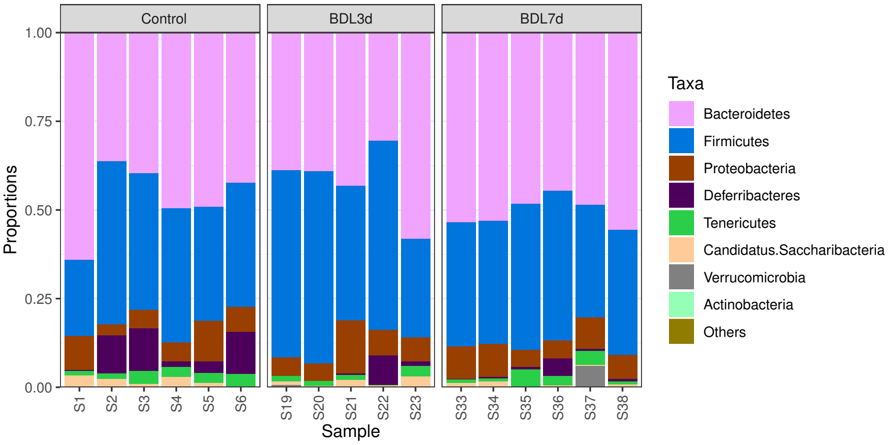
Control
BDL3d
BDL7d
1.00
0.75
0.50
0.25
0.00
Proportions
S1
S2
S3
S4
S5
S6
S19
S20
S21
S22
S23
S33
S34
S35
S36
S37
S38
Sample
Taxa
Bacteroidetes
Firmicutes
Proteobacteria
Deferribacteres
Tenericutes
Candidatus.Saccharibacteria
Verrucomicrobia
Actinobacteria
Others

B

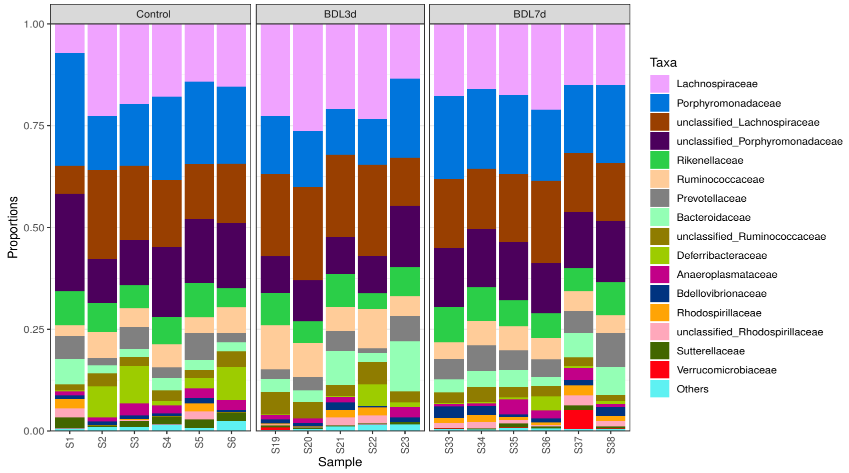
Control
BDL3d
BDL7d
1.00
0.75
0.50
0.25
0.00
Proportions
S1
S2
S3
S4
S5
S6
S19
S20
S21
S22
S23
S33
S34
S35
S36
S37
S38
Sample
Taxa
Lachnospiraceae
Porphyromonadaceae
unclassified_Lachnospiraceae
unclassified_Porphyromonadaceae
Rikenellaceae
Ruminococcaceae
Prevotellaceae
Bacteroidaceae
unclassified_Ruminococcaceae
Deferribacteraceae
Anaeroplasmataceae
Bdellovibrionaceae
Rhodospirillaceae
unclassified_Rhodospirillaceae
Sutterellaceae
Verrucomicrobiaceae
Others

Supplemental Figure legend

Supplemental Figure 1: Effects of BDL treatment on bacterial composition. (A) Phylum level bacterial composition in individual samples obtained from mice (control, BDL 3d and BDL 7d). **(B)** Family level bacterial composition in individual samples obtained from mice (control, BDL 3d and BDL 7d).
